# P68 RNA Helicase facilitates Breast Cancer progression by promoting Proliferation and Migration via PDGFR-β/AR axis

**DOI:** 10.7150/jca.61505

**Published:** 2021-09-09

**Authors:** Neha Panchbhai, Ravi Chakra Turaga, Malvika Sharma, Ganesh Satyanarayana, Zhi-Ren Liu

**Affiliations:** Department of Biology, Georgia State University, Atlanta, GA 30303, USA.

**Keywords:** P68 RNA helicase, Breast Cancer, PDGF receptor, Migration, Proliferation

## Abstract

Aberrant expression of P68 RNA helicase (p68), a prototypical member of the DEAD box family of RNA helicases, contributes to tumor development and progression. P68 tyrosine phosphorylation induced by PDGF signaling facilitates cancer metastasis by promoting EMT. In this report, we show that p68 promotes breast cancer cell EMT and cell migration by upregulation of PDGF receptor β (PDGFR-β). Knockdown of p68 in MDA-MB-231 and BT549 cells significantly decreases PDGFR-β both in mRNA and protein levels. P68 promotes EMT and cell migration in response to PDGF-BB stimulation via upregulation of PDGFR-β, suggesting that p68 enhances PDGF signaling by a positive feedback loop in cancer cells. Furthermore, our study reveals that p68 mediates the effects of PDGFR-β in regulation of androgen receptor (AR) in breast cancer cells. We demonstrate that p68 and PDGFR-β co-regulate AR expression and promote androgen-mediated proliferation in breast cancer cells. Our studies uncover an important pathway of p68-PDGFR-β axis in promoting breast cancer progression.

## Introduction

P68 RNA helicase (DDX5), a prototypic member of the DEAD box family of RNA helicases 1, is crucial for the cell development, division, and organ maturation 2. As an RNA helicase, ATPase and RNA unwinding activity of p68 have been well documented 3, 4. The previous reports also demonstrate the important role of p68 in diverse cellular processes, such as pre-mRNA, rRNA, miRNA processing, cell proliferation, and cell migration via epithelial mesenchymal transition (EMT) 5-7. Expression and post-translational modifications of p68 have been implicated in cancer development and progression 8.

Platelet derived growth factor receptor β (PDGFR-β) is a receptor tyrosine kinase that is implicated in cell proliferation upon PDGF-BB stimulation 9, 10. Evidences indicate an increase in PDGFR-β signaling in different stages of breast cancer and that it is highly upregulated in the human late stage mammary tumors. PDGFR-β expression has also been correlated with an invasive phenotype in human breast cancer 10. It has been established that an autocrine loop of platelet derived growth factor (PDGF-BB) and its cell surface receptor (PDGFR-β) is established in breast cancer cells through upregulation of both ligands and receptors, which results in cancer cell proliferation, migration, survival, and drug resistance 11-14.

Nuclear localization of PDGFR-β upon PDGF-BB stimulation has recently been reported. Nuclear interaction of PDGFR-β controls cell proliferation by chromatin remodeling and regulation of p21 levels 15. Also, nuclear PDGFR-β has been shown to control androgen receptor (AR) expression 15, 16. Notably, AR overexpression is associated with breast cancer aggressiveness 17. It has also been reported that p68 RNA helicase is a coactivator of various steroid hormone receptors, including AR 18. Clark et al. have previously demonstrated that p68 recruits AR and β-catenin to the promoter regions of androgen responsive genes, including prostate specific antigen resulting in increased AR transcriptional activity in prostate cancer 19.

Aberrant expression of p68 and PDGFR-β have been documented in three major human cancers, including breast cancer 20-23, colon cancer 24, 25, and prostate cancer 26, 27, suggesting the crucial role of both in tumorigenesis and cancer progression. Both p68 and PDGFR-β play an important role in cell proliferation and migration that is critical to cancer development. We have previously shown that p68 mediates PDGF-induced EMT by displacing axin from β-catenin 6. Here, we demonstrate that p68 also regulates PDGFR-β expression by a positive feedback loop in breast cancer cells. Upregulation of PDGFR-β by p68 results in enhanced EMT and migration of MDA-MB-231 and BT549 cells. Although, recent report has shed light on the translocation of PDGFR-β inside the nucleus, the role of the receptor nuclear localization in tumor progression is not well understood. Our study unveils a novel mechanism of AR regulation through p68-PDGFR-β axis. We show that p68 and nuclear PDGFR-β co-regulate AR and thereby mediate androgen dependent cell proliferation. Taken together, our findings reveal an important new mechanism of regulation through p68-PDGFRβ-AR axis, resulting in enhanced cancer cell proliferation and migration, and therefore progression of breast cancer.

## Materials and Methods

### Cell lines and treatments

Breast cancer cell lines MDAMB231, BT549, T47D, MCF7, HCC1806 were purchased from ATCC and were cultured per vendor's instructions. In all the experiments, cells were stimulated with 10 nM PDGF-BB unless otherwise stated in figures and legends. Treatment with PDGFR-β inhibitor, AG1296 (Cayman, 146535-11-7) at a concentration of 5 μM was used to inhibit PDGFR-β signaling.

### Transfection

To knock down p68 and PDGFR-β in MDAMB231 and BT549 cells, siRNA p68 (SCBT, sc-37141) or siRNA PDGFR-β (SCBT- sc29442) respectively was transfected with 10 nM of siRNA for 48 h using Lipofectamine™ RNAiMAX Transfection Reagent kit (Thermo Fisher Scientific). To overexpress PDGFR-β in MDAMB231 and BT549 cells, 2.5 μg of PDGFR-β plasmid (Origene RC206377) was transfected for 48 h using Lipofectamine™ 3000 Transfection Reagent kit (Thermo Fisher scientific).

### Immunoblotting

The proteins were analyzed by immunoblotting probed with antibodies against p68 (SCBT, sc-126730), PDGFR-β (Abclonal, Ab2180), phospho-PDGFR-β (SCBT, Sc365465), phospho-EGFR (Abclonal, Ab 40815), EGFR (Cell signaling, 26465), FGFR (SCBT, sc-390423), N-Cadherin (Invitrogen, 33-3900), E-Cadherin (Biosciences, 610404), Vimentin (Protein tech, 60330), Snail (Abcam, 17732), AR (Agilent, M356201), β-Actin (2bscientific, R15006MC4) according to the vendor's instructions.

### Scratch Assay

MDAMB231 and BT549 cells were cultured and transfected with control siRNA, p68 si RNA, PDGFR-β plasmid or co-transfected with p68 siRNA and PDGFR-β plasmid for 48 h in the presence of 10 nM PDGF-BB. Multiple scratches were introduced in the cultured plates. The scratch treated cells were directly visualized under the microscope.

### Boyden chamber assay

Falcon® Permeable Support, 24 well plate with 8.0 μm transparent PET membrane chambers (Life sciences) was used to measure the migration of MDAMB231 and BT549 cells. The test cells were first transfected with either control siRNA or p68 siRNA (SCBT, sc-37141) for 48 h in regular cell culture plates. The treated cells were re-suspended into optimum medium (without serum) and seeded onto the PET membrane chamber. The DMEM culture medium with 10% FBS was added to the outer chambers. After 12 h incubation, medium in the inner chamber was removed and the cells attached to the outer bottom side were fixed with paraformaldehyde and stained with 0.5% crystal violet. The stained cells were imaged under the inverted microscope.

### Immunohistochemistry

Breast cancer array (US Biomax, BC08118a) was deparaffinized and incubated in Lab Vision Hydrogen peroxide block and blocked with Lab Vision Protein block reagent. Tissue array was incubated with rabbit monoclonal antibody against p68 (1:500 dilution) for 1 h at room temperature followed by detection with the Horseradish Peroxidase- 3,3′-Diaminobenzidine (DAB). Nuclear counterstaining was done using hematoxylin.

### Immunofluorescence

Cells were fixed in paraformaldehyde and blocked with 5% goat serum at room temperature for 1 h. The cells were incubated with PDGFR-β (1:200) antibody in a humidified chamber overnight. Anti-rabbit secondary antibody conjugated with Alexa Fluor-555 was used to label PDGFR-β. Nuclei were stained and mounted with DAPI (Life technologies, S36938). Images were visualized in a confocal microscope.

### Real time-PCR

RNA isolation was performed by generic Trizol-phenol chloroform protocol. The isolated RNA was quantified using Nanovue plus™ spectrophotometer (Biochrom, 28956057). cDNA was prepared using the Thermo scientific C-DNA kit. Real time PCR was performed using Luna Universal qPCR master mix (M3003L, NEB Inc) according to the vendor's instructions. The primer for the genes are: DDX5 (F- GCCGACGTCAACGGGAAAGT, R-CGTTGTCACGTGGCTGTACC), PDGFR-β (F-CCCCTTTCTGGCCTGATGCTC, R-TGATGTTCTCACCCTGGCGG), AR (F-GAAAGCGACTTCACCGCACC, R-AGGAATCCCTCTCTGCTCGC), Vimentin (F-TCCGCACATTCGAGCAAAGA, R-ATTCAAGTCTCAGCGGGCTC), N-cadherin (F-GCCAGAAAACTCCAGGGGAC, R-TGGCCCAGTTACACGTATCC), Snail (F-GCTGCAGGACTCTAATCCAGA, R-ATCTCCGGAGGTGGGATG), E-cadherin (F-TCATGAGTGTCCCCCGGTAT, R-TCTTGAAGCGATTGCCCCAT), EGFR (F-GGCAGGAGTCATGGGAGAA, R-GCGATGGACGGGATCTTAG), FGFR (F-ATGGCAACCTTGTCCCTG, R-CAGCGCACCTCTAGCGAC), c-FOS (F- GTGTCCACCGCTGCCTTC, R- GCCACCATTGCTGAAGGGATT). The primers were designed using NCBI Blast. Quantitative PCR was done under the following thermocycler conditions: 95 °C for 2 min, and 40 cycles of 95 °C for 30s, 62 °C for 1 min, and 95 °C for 30 s. Data was collected from 7500 Fast Real-Time PCR System (Applied Biosystems).

### MTT assay

MDAMB231 and BT549 cells transfected with control siRNA or p68 siRNA were treated with or without DHT (1 nM) for 24 h. To analyze the cell proliferation, these treated cells were incubated with Thiazolyl Blue Tetrazolium Bromide (MTT) (Millipore sigma, M5655) for 4 h at 37 °C. The absorbance was measured at 595 nm.

### Kaplan-Meier survival

KM plotter (www.kmplot.com) was used to analyze the correlation of DDX5 mRNA expression to the overall survival (OS) of breast cancer patients. Log rank *p*-value and hazard ratio (HR) with 95% confidence intervals were calculated and displayed on the webpage.

### Statistical analysis

To compare the two appropriate groups, the data sets were statistically analyzed. The *P* values were calculated using unpaired two-tailed Student's *t*-test or ANOVA test. NS denotes non-significant: *P*>0.05, *P<0.05, **P<0.01, ***P<0.001, and ****P<0.0001.

## Results

### Elevation in p68 expression is associated with low overall survival in breast cancer patients

**Figure 1:**
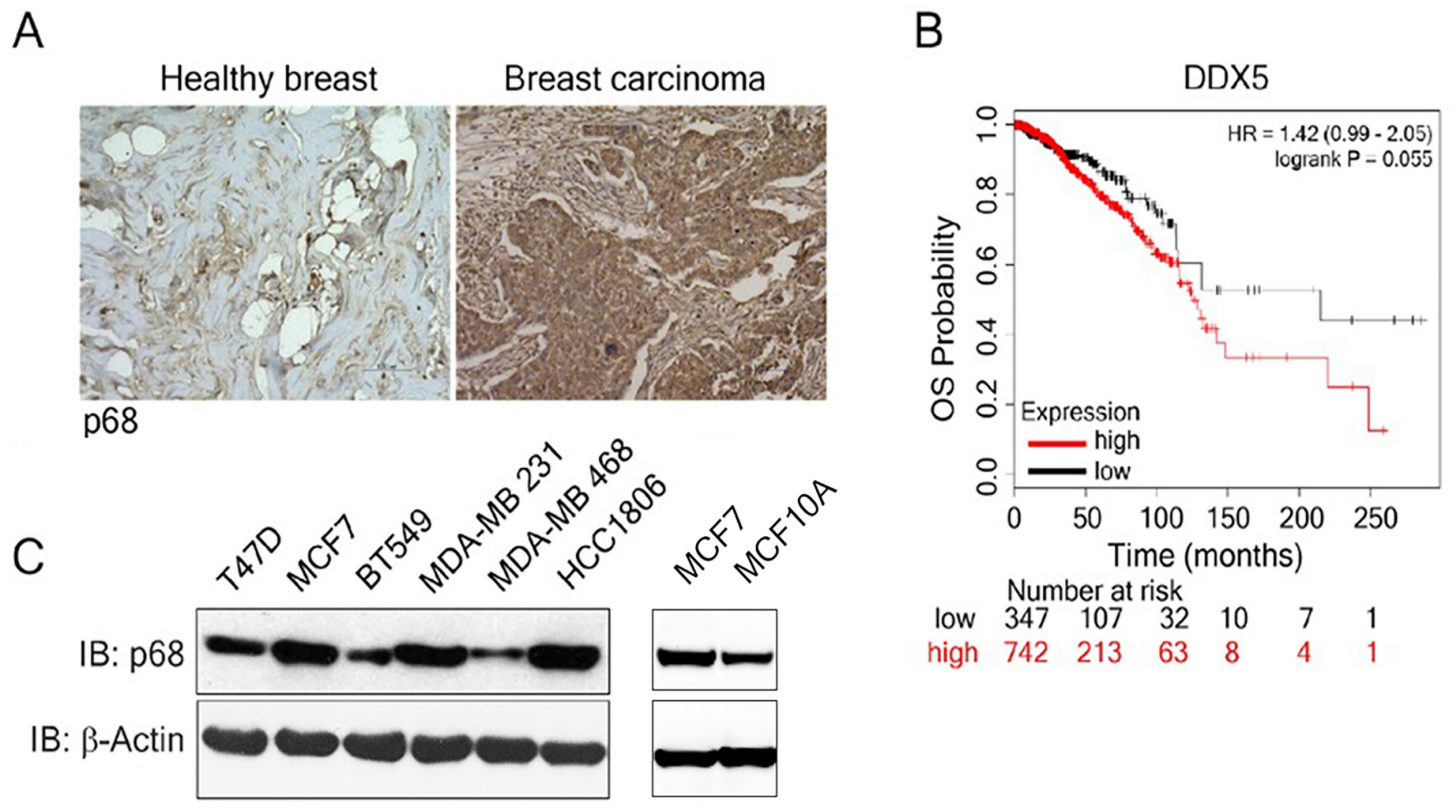
** Overexpression of p68 in tumor is associated with low overall survival in breast cancer patients. (A)** Representative images of immunohistochemistry staining of p68 in healthy breast and breast tumor tissue of patients. **(B)** Kaplan-Meier survival analysis of low and high DDX5 gene expression (DDX5 low (n=347); DDX5 high (n=742) in the tumor of breast cancer patients. **(C)** Levels of p68 (IB: p68) in the indicated breast cancer cell lines and MCF-10A cells were analyzed by immunoblot. Immunoblot of β-actin (IB: β-actin) is the loading control.

We examined p68 levels by immunostaining the breast carcinoma patient tissue array (n=60), as well as the normal breast tissue (n=3). The expression of p68 displayed marked elevation in the breast carcinoma tissue when compared to the normal healthy breast tissue (Fig. 1A). Further, we analyzed the impact of p68 expression on the overall survival (OS) in breast cancer patients using publicly available data set 28. High expression of p68 closely correlates with low OS in patients with breast cancer (Fig. 1B). We then analyzed p68 levels in multiple breast cancer and normal cell lines and found that p68 is highly expressed in the tested breast cancer cell lines, but expression levels in normal cells is lower (Fig. 1C). Altogether, our results suggest that p68 is elevated in breast cancer, and the expression of p68 may play a critical role in breast cancer progression.

### P68 RNA helicase mediates PDGF signaling in promoting EMT and cell migration of breast cancer cells

**Figure 2:**
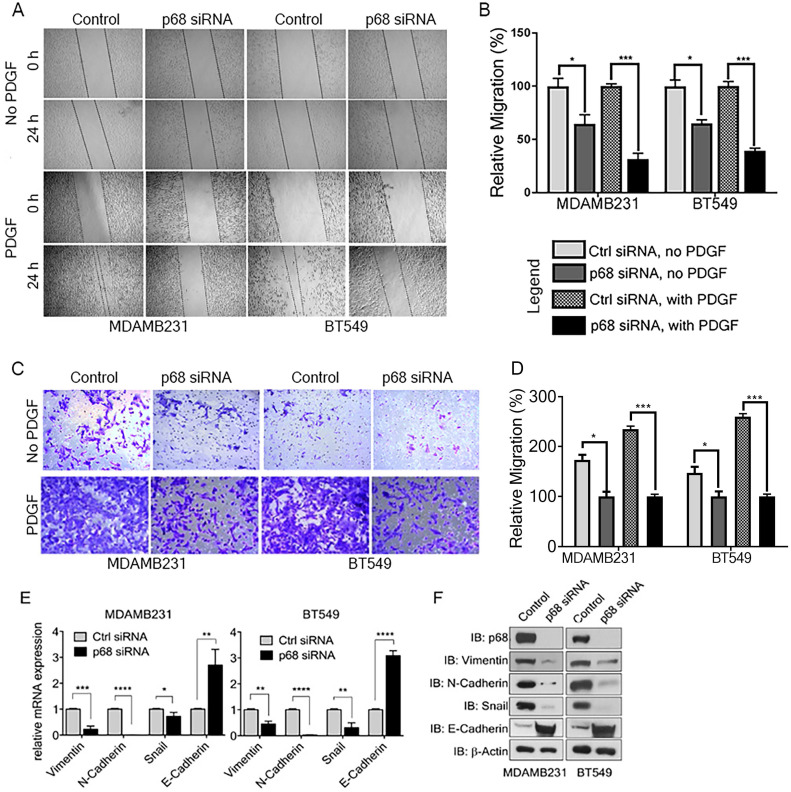
** P68 RNA helicase knockdown decreases migration of breast cancer cells in response to PDGF-BB stimulation. (A-D)***In vitro* scratch wound healing assay (A) and Boyden chamber assay (C) of MDAMB231 (left panel) and BT549 (right panel) cells upon p68 knockdown in response to PDGF-BB stimulation compared with control siRNA and quantitative analyses of the migrating cells (B, D). In A-D, No PDGF means the cells were not treated by PDGF-BB. **(E & F)** Relative mRNA expression (E), and immunoblot analysis (F) of EMT markers such as vimentin (IB:vimentin), N-cadherin (IB:N-cadherin), Snail (IB:Snail), and E-cadherin (IB:E-cadherin) in PDGF-BB (20 ng/ml, 24h) stimulated MDAMB231 and BT549 cells upon p68 knockdown compared with control siRNA. Knockdown efficiency of p68 (IB:p68) was analyzed by immunoblot. Immunoblot of β-actin (IB: β-actin) is the loading control. Error bars represent mean ± S.E.M. *P<0.05, **P<0.01, ***P<0.001, ****P<0.0001.

We previously reported that tyrosine phosphorylation of p68 mediates the effects of PDGF-induced EMT via promoting β-catenin nuclear translocation 6. P68 and Ca^2+^-calmodulin interaction promotes cells migration 29. We assessed the basal expression of PDGFR-β in breast cancer cell lines, including T47D, MCF-7, BT549, MDA-MB-231, MDA-MB-468, and HCC 1806 by immunoblotting. The tested breast cancer cell lines express PDGFR-β (Supplementary Fig. S1A). To elucidate the role of p68 in breast cancer cells migration stimulated by PDGF, we performed scratch-wound and Boyden chamber assay with two highly invasive breast cancer cell lines, MDA-MB-231 and BT549. We first knocked-down endogenous p68 by RNAi in the cells. Both Boyden chamber and scratch wound assay showed that p68 knockdown significantly reduced cell migration upon PDGF-BB stimulation when compared with scrambled siRNA (Fig. 2A-D). Interestingly, we noted clear morphological differences with or without p68 knockdown upon PDGF-BB stimulation. These morphological differences clearly indicated the role of p68 in epithelial-mesenchymal transition (EMT) upon PDGF stimulation (Supplementary Fig. S1B). To validate the observation, we examined the expression of EMT markers upon PDGF stimulation in p68 knockdown cells. The mesenchymal markers, including vimentin, N-cadherin, and snail, were significantly reduced both in mRNA (Fig. 2E) and protein (Fig. 2F) levels upon PDGF treatment in p68 knockdown cells, while the epithelial markers E-cadherin were significantly increased both in mRNA (Fig. 2E) and protein (Fig. 2F) levels upon PDGF treatment. The results indicate that p68 mediates the effects of PDGF-BB in promoting breast cancer cell EMT and migration.

### P68 RNA helicase regulates PDGFR-β expression in breast cancer cells

**Figure 3:**
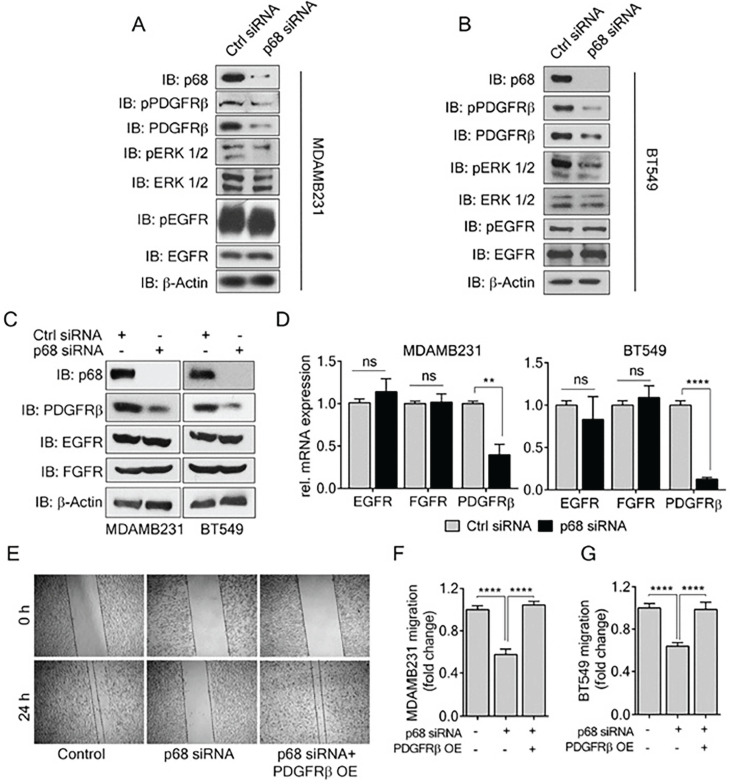
** P68 RNA helicase transcriptionally regulates PDGFR-β expression in breast cancer cells. (A, B)** Levels of p-PDGFRβ (IB: p-PDGFRβ) and pERK ½ (IB:pERK1/2), an established effector of PDGFR-β signaling in MDAMB231 (A) and BT549 (B) cells were analyzed by immunoblot upon PDGF-BB (20 ng/ml, 24h) stimulation. Levels of ERK ½ (IB:ERK1/2) and PDGFR-β (IB: PDGFRβ) are the loading controls. **(C, D)** Immunoblot analysis (C) of PDGFR-β (IB: PDGFR-β), EGFR (IB:EGFR), and FGFR (IB:FGFR) and their relative mRNA expression (D) in PDGF-BB stimulated MDAMB231 and BT549 cells upon p68 knockdown. Knockdown efficiency of p68 (IB:p68) was analyzed by immunoblot. Immunoblot of β-actin (IB:β-actin) is a loading control. **(E)** *In vitro* scratch wound healing assay of MDAMB231 cells transfected with p68 siRNA or co-transfected with p68 siRNA and PDGFRβ plasmid compared with control siRNA at 0 h and 24 h upon PDGF-BB stimulation. **(F & G)** Quantitative analyses of the migrating PDGF-BB stimulated MDAMB231 (F), and BT549 (G). The results represent findings of five independent experiments. Error bars represent mean ± S.E.M. **P<0.01, ****P<0.0001, ns denotes non-significant.

PDGF-BB induces migration and invasion of cancer cells, including breast cancer cells. Previous reports have shown that PDGFR-β overexpression correlates with invasiveness of breast cancer 10. Since we observed a decrease in EMT and migration in p68 knockdown cells upon PDGF-BB treatment, we sought to investigate the effects of p68 knockdown on PDGF-BB signaling, probing the levels of phospho-PDGFR-β in the p68 knockdown breast cancer cells upon PDGF-BB stimulation. In addition to decrease in phospho-PDGFR-β, we also observed a decrease in PDGFR-β levels in both MDA-MB-231 and BT549 cells (Fig. 3A & B). In consistent, we found that p68 knockdown also led to a reduction in mRNA of PDGFR-β, a two-fold decrease in MDA-MB-231 cells and a ten-fold decrease in BT549 cells compared with the scrambled siRNA controls (Fig. 3D). To determine whether the effect of p68 on intracellular PDGFR-β and phosphor-PDGFR-β is specific, we measured the levels of c-FOS, a PDGFR-β responsive gene, upon p68 knockdown. The mRNA levels of c-FOS were decreased both in p68 knockdown MDA-MB-231 and BT549 cells upon PDGF-BB treatment (Supplementary Fig. S2). However, p68 knockdown did not alter the phosphorylation and expression of other growth factor receptors, including EGFR and FGFR, in the breast cancer cells (Fig. 3A-C). Thus, our data indicates that p68 plays a role in regulating PDGFR-β expression in response to PDGF stimulation in breast cancer cells. We reasoned whether the effect of p68 knockdown on the cell migration is due to the reduction in PDGFR-β expression. To test this conjecture, we performed scratch wound assay with MDA-MB-231 and BT549 cells in which p68 was knocked down and PDGFR-β was exogenously expressed in the p68 knockdown cells. Evidently, p68 knockdown reduced cell migration under PDGF-BB treatment. Exogenous expression of PDGFR-β recovered the migration reduction of the cells in which p68 was knocked down (Fig. 3E-G). Furthermore, we tested migration of MDA-MB-231 cells in which PDGFR-β was knocked down or the receptor was exogenously expressed with or without p68 knockdown. Knockdown of p68 or PDGFR-β (Supplementary Fig. S3C) decreased migration of MDA-MB-231 cells. Exogenous overexpression of PDGFR-β relieved the effects of p68 knockdown on MDA-MB-231 cells migration (Supplementary Fig. S3A). It was also noted that exogenous overexpression of PDGFR-β restored the fibroblast-like morphology of p68 knockdown MDA-MB-231 and BT549 cells (Supplementary Fig. S1B). We further examined the expression of EMT markers, including vimentin, snail, and E-cadherin, to validate the observation. Consistent with our previous observation, a decrease in vimentin/snail and an increase in E-cadherin were observed upon p68 knockdown. Exogenous expression of PDGFR-β in p68 knockdown MDA-MB-231 and BT549 cells increased levels of vimentin, snail and decreased E-cadherin (Supplementary Fig. S3B). Our findings suggest that p68 mediates PDGF-BB signaling in promoting EMT and cell migration by regulation of PDGFR-β expression.

### P68 and PDGFR-β co-regulate AR expression and mediate Dihydrotestosterone (DHT)-induced proliferation in breast cancer cells

**Figure 4:**
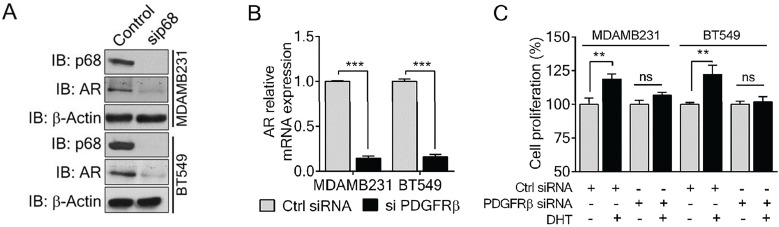
** P68 RNA helicase regulates AR expression and mediates the effect of DHT in proliferation of breast cancer cells. (A & B)** Immunoblot analysis (A), and relative mRNA expression (B) of AR in PDGF-BB stimulated MDAMB231 and BT549 cells upon p68 knockdown. Knockdown efficiency of p68 (IB: p68) was analyzed by immunoblot. Immunoblot of β-actin (IB: β-actin) is the loading control**. (C)** Cell proliferation of MDAMB231 and BT549 cells untreated (open bar) or treated (filled bar) with DHT (1 nM for 24 h) upon p68 knock down compared with the control. The experiments were performed in triplicate. Error bars represent mean ± S.E.M. *P<0.05, **P<0.01, ***P<0.001, ns denotes non-significant.

**Figure 5:**
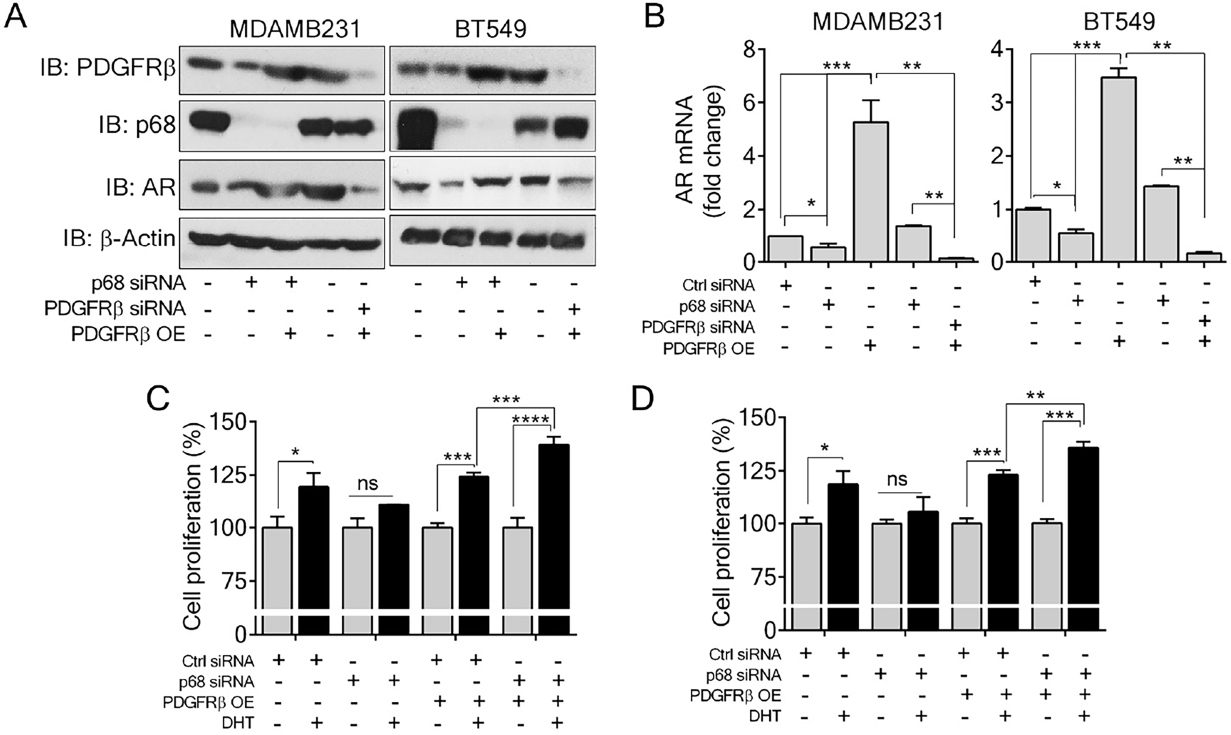
** PDGFR-β regulates AR expression and mediates the effect of DHT in proliferation of breast cancer cells. (A & B)** Immunoblot analysis (A), and relative mRNA expression (B) of AR in PDGF-BB stimulated MDAMB231 and BT549 cells upon PDGFR-β knockdown. Knockdown efficiency of PDGFR-β (IB: PDGFR-β) was analyzed by immunoblot. β-actin (IB: β-actin) is the loading control. **(C)** Cell proliferation of MDAMB231 and BT549 cells untreated (open bar) or treated (filled bar) with DHT (1 nM for 24 h) upon PDGFR-β knock down compared with the control. The experiments were performed in triplicate. Error bars represent mean ± S.E.M. **P<0.01, ***P<0.001, ns denotes non-significant.

P68 RNA helicase has been demonstrated as an AR transcriptional coactivator 26. On the other hand, PDGFR-β controls AR expression 16. Importantly, AR is commonly expressed in breast cancers and is associated with poor prognosis of patients 17. Therefore, we sought to investigate whether p68 and PDGFR-β co-regulate AR expression in breast cancer cells. We first validated that p68 knockdown in both MDA-MB-231 and BT549 cells resulted in reduction in AR in the cells both in protein and mRNA levels (Fig. 4A & B). It has been demonstrated that tyrosine phosphorylation plays an important role in PDGF-BB induced cell proliferation via upregulation of cyclin D1 and c-Myc 7. It is well established that androgen [dihydrotestosterone (DHT)] treatment promotes cell proliferation in prostate cancer and breast cancer 30, 31. We investigated the effects of p68 on cell proliferation under treatment of PDGF-BB and DHT. We observed that there was an increase in cell growth rate of MDA-MB-231 and BT549 cells upon DHT treatment in addition to PDGF-BB stimulation. However, the growth rate increase by DHT treatment was diminished by p68 knockdown (Fig. 4C). Clearly, p68 regulates AR expression and consequently the function of AR in promoting breast cancer cell proliferation in response to androgen stimulation. PDGFR-β has also been shown to control AR expression. Therefore, we further investigated AR expression in PDGFR-β knockdown breast cancer cells. We found a significant reduction in both protein (Fig. 5A) and mRNA (Fig. 5B) levels of AR upon PDGFR-β knockdown both in MDA-MB-231 and BT549 cells. Cell proliferation was significantly inhibited in the PDGFR-β knockdown cells upon DHT treatment (Fig. 5C & D). Our results suggest that both p68 and PDGFR-β regulate AR expression and control androgen dependent proliferation in breast cancer cells.

Since p68 regulates PDGFR-β expression, we sought to test whether exogenous expression of PDGFR-β in p68 knockdown cells would alter the cellular AR levels. Interestingly, expression of PDGFR-β in p68 knockdown breast cancer cells restored AR expression both in protein (Fig. *5*A) and mRNA (Fig. *5*B) levels, suggesting that p68 may regulate AR expression via PDGFR-β. Cell proliferation assay further corroborated our finding that exogenous expression of PDGFR-β in p68 knockdown MDA-MB-231 and BT549 cells resulted in an increase in cell proliferation upon DHT treatment (Fig. 5C & D). Taken together, our data suggest that p68 and PDGFR-β co-regulate AR expression and mediate androgen dependent proliferation in breast cancer cells.

### Nuclear PDGFR-β regulates AR expression in breast cancer cells

**Figure 6:**
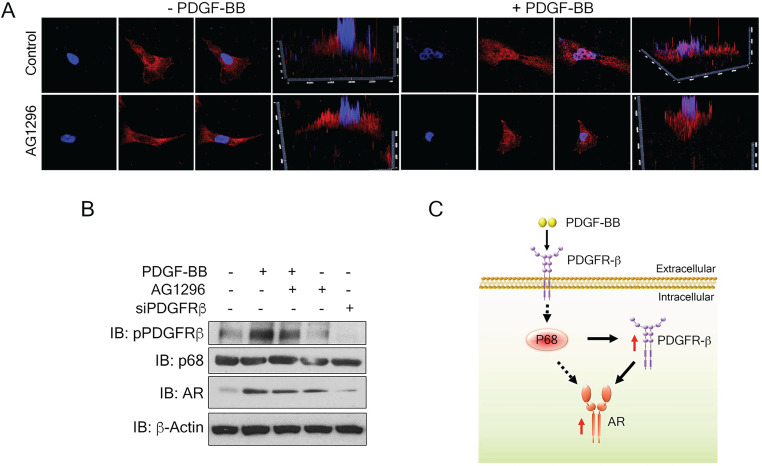
** Nuclear PDGFR-β regulates AR expression in breast cancer cells. (A)** Representative images of immunofluorescence staining of PDGFR-β (red) MDAMB231 cells treated with PDGFR-β inhibitor, AG1296 with or without PDGF-BB stimulation for 24 h compared with the control. Nuclei were stained with Hoescht (blue). **(B)** Levels of AR (IB: AR) in MDMB231 cells upon indicated conditions were analyzed by immunoblot. Levels of pPDGFR-β (IB: pPDGFR-β), PDGFR-β (IB:PDGFR-β) and p68 (IB:p68) are the controls, indicating the efficiency of AG1296, PDGFR-β knockdown efficiency, and total p68 respectively. Immunoblot of β-actin (IB:β-actin) is the loading control. **(C)** Schematic illustration of p68- PDGFRβ-AR axis in breast cancer cells upon PDGF-BB stimulation.

To understand the mechanism by which PDGFR-β regulates AR expression in breast cancer cells, we examined the levels of PDGFR-β in MDA-MB-231 cells upon PDGF-BB stimulation. High nuclear PDGFR-β was observed in the treated cells (Fig. 6A), which is consistent with the observation by Papadopoulos et al. that PDGFR-β translocates to the cell nucleus in response to PDGF-BB stimulation. Notably, the nuclear PDGFR-β plays an important role in controlling cell proliferation. In addition, PDGFR-β kinase activity is not essential for nuclear accumulation of the receptor 15 To validate whether the receptor kinase activity is required for PDGFR-β nuclear localization, we examined the nuclear PDGFR-β upon addition of phospho-PDGFR-β inhibitor, AG1296. In response to PDGF-BB stimulation, the nuclear PDGFR-β levels did not experience any significant change upon AG1296 treatment (Fig. 6A & B). We analyzed the AR levels in the PDGF-BB stimulated cells in the presence of AG1296. Clearly, AG1296 treatment did not change the AR levels in the cells (Fig. 6B). Thus, our results indicate that nuclear PDGFR-β regulates AR expression in breast cancer cells.

## Discussion

P68 RNA helicase aberrant expression is associated with poor prognosis of patients and breast cancer progression 21. We have previously reported that p68 promotes cell migration and proliferation via Wnt/*β*-catenin signaling pathway 6. In this study, we report that p68 regulates expression of the growth factor receptor, PDGFR-β in response to PDGF-BB stimulation, and thereby promotes EMT and enhances migration of breast cancer cells. The role of p68 in regulation of PDGFR-β expression under stimulation of the growth factor forms an important positive feedback loop in facilitating breast cancer progression via promoting EMT and cell migration. In addition, p68 cooperates with PDGFR-β to co-regulate AR expression, therefore facilitates androgen dependent proliferation in breast cancer cells. Both PDGFR-β and AR are very important prognostic markers for breast cancer patients 32. Apparently, p68 plays a critical role in coordinating both PDGF and androgen signaling in breast cancer progression.

P68 has been demonstrated as a co-activator of AR 18. Androgen receptor is highly expressed in invasive breast cancer and prostate cancer cells. A recent report highlights the androgen-mediated invasiveness of triple negative breast cancer (TNBC) through AR/Src/PI3-K complex 33. Androgen-induced proliferation via androgen receptor has also been well documented. Additionally, PDGFR-β has been shown to control AR expression 16. Because p68 siRNA decreases the levels of AR, and p68 regulates PDGFR-β upon PDGF stimulation, we therefore believe that there is regulatory axis of p68-PDGFR-β-AR in PDGF-BB and androgen signaling, wherein p68 and PDGFR-β co-regulate AR expression in breast cancer cells. This regulatory axis mediates and coordinates the effects of both androgen and PDGF-BB signaling in promoting breast cancer progression (Fig. 6C). How p68 controls these two gene expressions remains an open question. P68 regulates the transcription of numerous genes, including Snail 1 34, androgen receptor (AR) 18. It has also been reported that p68 interacts with histone deacetylase 1, RNA polymerase II holoenzyme, and p300/CBP, suggesting its function in transcription 35, 36. A reasonable speculation is that, under PDGF-BB stimulation, p68 may be phosphorylated. The phosphorylated p68 translocates to the cell nucleus and acts on PDGFR-β transcription. Nuclear p68 may also act as a co-activator for PDGFR-β in AR transcription. PDGFR-β and AR inhibition have been considered as approaches for cancer therapy. However, neither inhibition of PDGFR-β nor AR has been successful in the clinic so far. The cooperation between p68 and PDGFR-β in regulation of AR may potentially be a reason for the failure.

## Supplementary Material

Supplementary figures.
