# Supplementary material for: P68 RNA Helicase facilitates Breast Cancer progression by promoting Proliferation and Migration via PDGFR-β/AR axis
Source: J Cancer. 2021 Sep 9;12(21):6543–52. doi: 10.7150/jca.61505 (PMC8489147; doi:10.7150/jca.61505)

On-line supplementary materials

P68 RNA Helicase Facilitates Breast Cancer Progression by Promoting Proliferation and Migration via PDGFR-β/AR Axis.

Neha Panchbhai[#], Chakra R. Turaga[#], Malvika Sharma[#], Ganesh Satyanarayana, and Zhi-Ren Liu[*]

Department of Biology, Georgia State University, Atlanta, GA 30303, USA

Supplementary Figure S1. (A) Expression of PDGFR-β (IB:PDGFR-β) in indicated breast cancer cell lines was analyzed by immunoblot. β-actin (IB:β-actin) is the loading control. **(B)** Representative images of phase contrast microscopy of PDGF-BB stimulated MDAMB231 cells upon indicated conditions.

Supplementary Figure S2. Relative mRNA expression of c-FOS, a PDGFR-β responsive gene in MDAMB231 and BT549 cells stimulated with PDGF-BB upon p68 knockdown. Error bars represent mean ± S.E.M. $^{**}P<0.01$, $^{***}P<0.001$ by unpaired Student's t-test.

Supplementary Figure S3. (A) Quantitative analyses of migrating MDAMB231 cells in the scratched areas upon indicated conditions. The results represent findings of three independent experiments. **(B)** Immunoblot analyses of EMT markers such as E-Cadherin, Snail, and Vimentin in PDGF-BB stimulated MDAMB231 and BT549 cells upon indicated conditions. Knockdown efficiency of p68, PDGFR-β, and PDGFR-β overexpression was analyzed by immunoblot of p68 and PDGFR-β.

(C) Immunoblot analysis of AR and PDGFR-β in PDGF-BB stimulated MDAMB231 and BT549 cells upon PDGFR-β knockdown. Knockdown efficiency was analyzed by immunoblot. In A and C, immunoblot of β-actin (IB: β-actin) is a loading control. Error bars represent mean ± S.E.M. $^{**}P<0.01$, $^{****}P<0.0001$, ns denotes non-significant by unpaired Student's t-test.

Supplementary Figure S4. Representative images of immunofluorescence staining of PDGFR-β (red) in MDAMB231 cells stimulated with PDGF-BB upon p68 knockdown compared with the control. Nuclei were stained with DAPI (blue).

Supplementary Figure S1

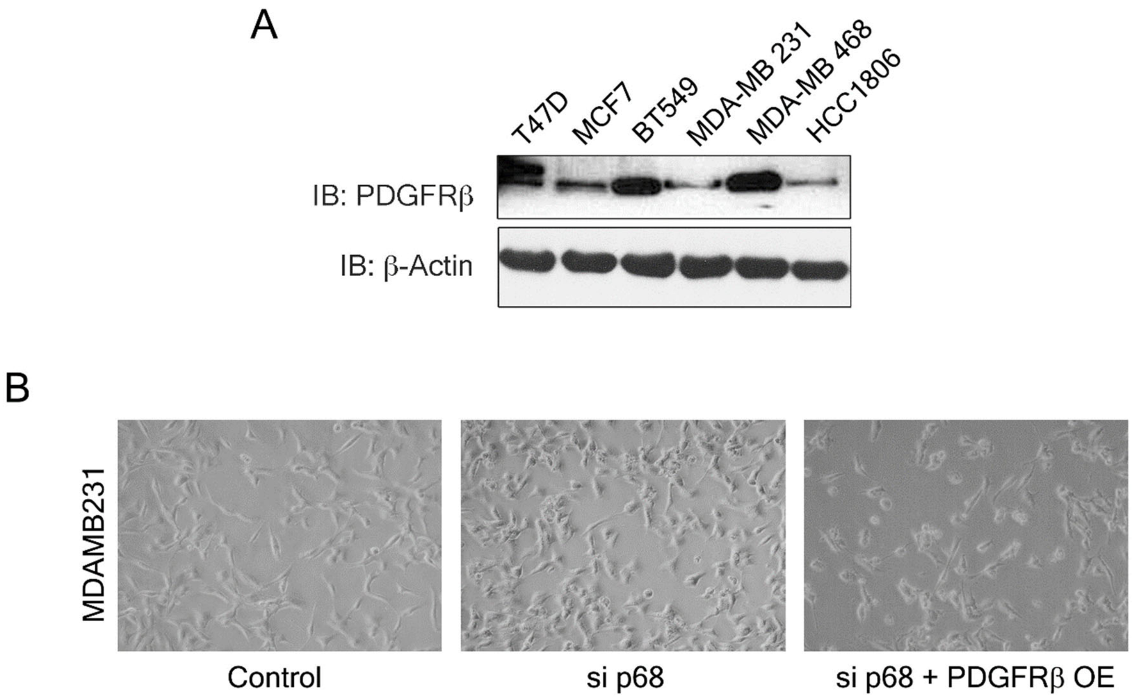

Supplementary Figure S2

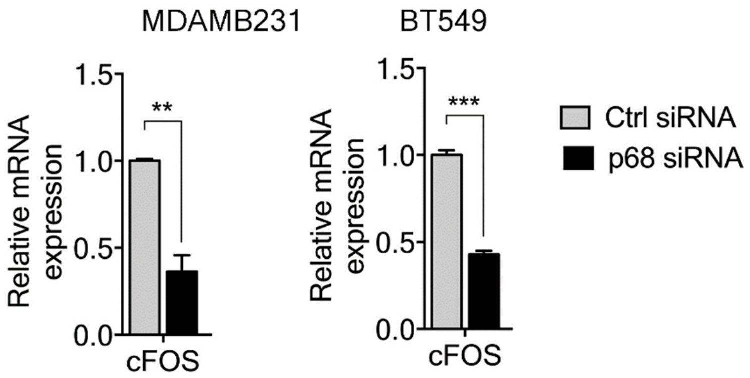

Supplementary Figure S3

A

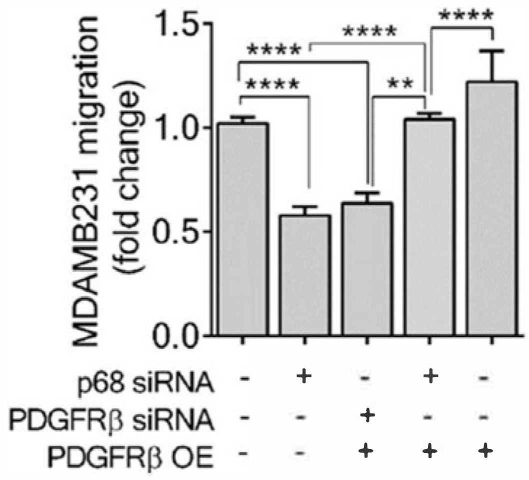

B

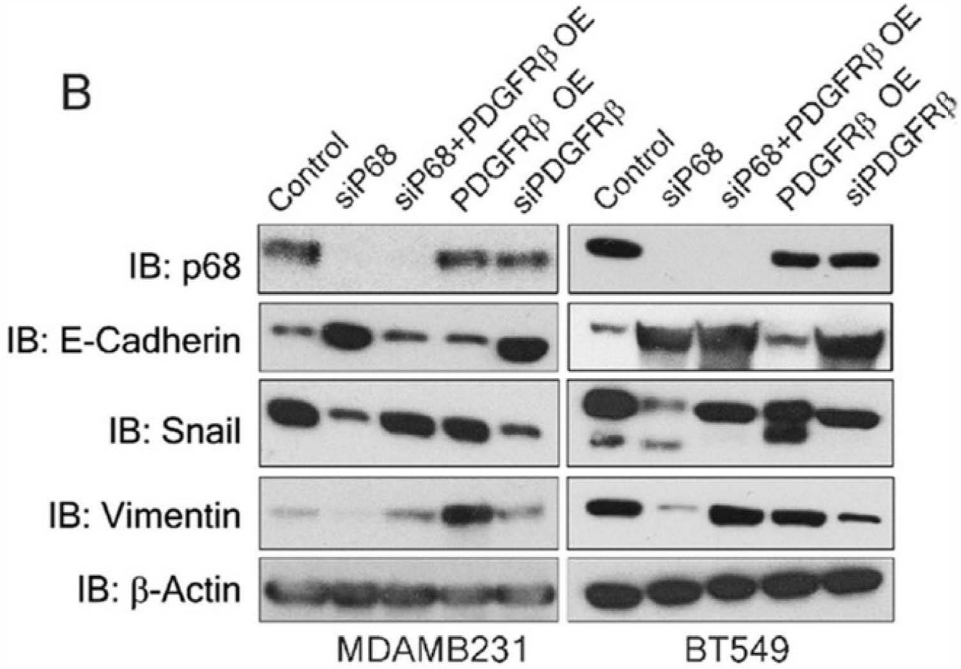

C

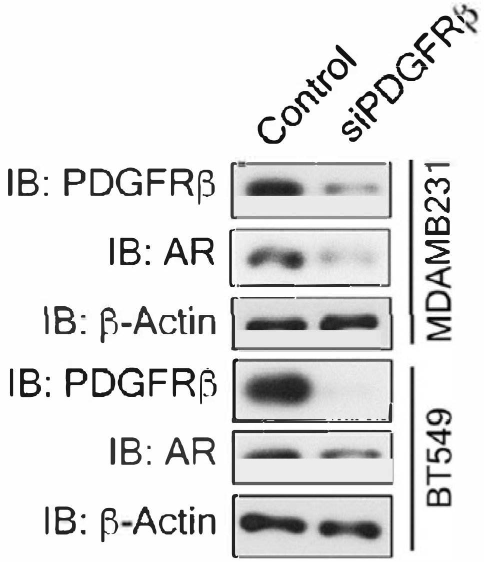

Supplementary Figure S4

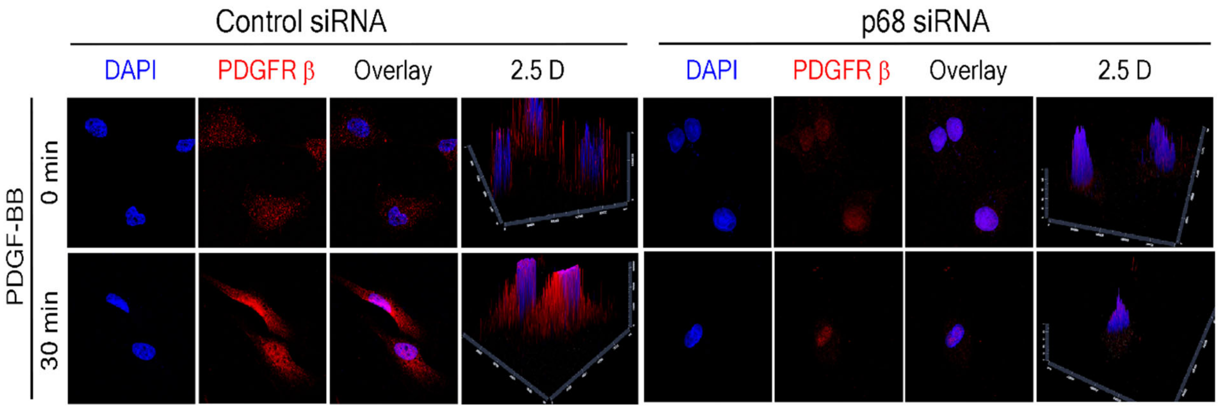

Supplement: Supplementary file 1 — Supplementary figures. [file jcav12p6543s1.pdf]
